# Personalized 3D‐Printed Affinisol‐Based Pharmaceutical Delivery Systems: The Case Study for Alternative Sulfonamides for Alzheimer's Disease

**DOI:** 10.1002/mabi.70266

**Published:** 2026-09-25

**Authors:** Marie Nevyhoštěná, Alena Komersová, Roman Svoboda, Vladimír Pejchal, Adéla Pospíšilová, Jan Muselík, Aleksandra Łapa, Paulina Koczurkiewicz‐Adamczyk

**Affiliations:** ^1^ Faculty of Chemical Technology University of Pardubice Pardubice Czech Republic; ^2^ Faculty of Pharmacy Masaryk University Brno Czech Republic; ^3^ Faculty of Pharmacy Jagiellonian University Medical College Kraków Poland

**Keywords:** Alzheimer's disease, dissolution testing, FDM 3D printing, hot melt extrusion, personalized medicine

## Abstract

The use of hot melt extrusion combined with fused deposition modeling 3D printing was explored to enhance the solubility and bioavailability of poorly water‐soluble compounds intended for Alzheimer's disease (AD) therapy. AD, a neurodegenerative disorder with no definitive treatment, affects millions worldwide, underscoring the urgent need for effective and personalized therapeutic strategies. Four sulfonamide‐derived compounds, identified as potential AD therapeutics, were synthesized and successfully incorporated (loads between 10 and 50 wt.%) into customized, 3D‐printed oral dosage forms. These 3D‐printed tablets were evaluated for properties such as hardness, thermo‐analytical characterization, dissolution, cytotoxicity, and stability. Notably, successful amorphization of the incorporated APIs was observed in most 3D‐printed tablets, which may be critical for improving solubility. In vitro dissolution testing revealed multiple favorable release profiles, with several formulations demonstrating sustained or enhanced drug release suitable for the therapeutic requirements of AD. When compared to the dissolution data of the corresponding conventional matrix tablets, the present 3D‐printed tablets offered superior or comparable dissolution characteristics, demonstrating the potential of personalized medicine and remote digital control for optimizing patient‐specific treatments.

## Graphical abstract

Personalized dosage forms engineered via hot‐melt extrusion and FDM 3D‐printing enable the evaluation of novel anti‐Alzheimer's compounds. The study integrates dissolution testing, thermo‐analytical profiling, physico‐chemical characterization, cytotoxicity, and stability to advance personalized medicine in drug development.

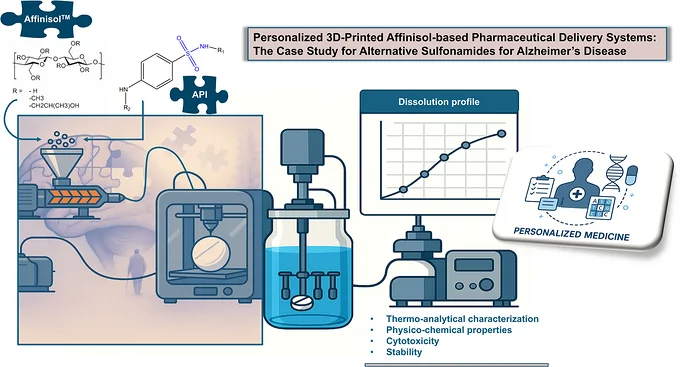

**Abbreviations**
AChE: acetylcholinesterase;
AD: Alzheimer's disease;
API: active pharmaceutical ingredient;
BChE: butyrylcholinesterase;
DMA: dynamic mechanical analysis;
DSC: differential scanning calorimetry;
FDM: fused deposition modeling;
HepG2: human hepatocellular carcinoma;
HME: hot melt extrusion;
HPMC: hydroxypropylmethylcellulose;
MC: moisture content;
MIC: minimum inhibitory concentration;
MTT: 3‐(4,5‐dimethylthiazol‐2‐yl)‐2,5‐diphenyltetrazolium bromide;
RIV: rivastigmine;
SH‐SY5Y: neuroblastoma cell line;
TGA: thermogravimetric

## 1 Introduction

Sulfonamides, widely used as antibacterial agents, also exhibit anti‐inflammatory and anticancer properties. They function as antioxidants and carbonic anhydrase inhibitors, making them relevant in the treatment of Alzheimer's disease (AD), a neurodegenerative disorder marked by cognitive decline and dementia [1]. Neuropathologically, AD is characterized by brain tissue loss due to massive neuronal necrosis, particularly in the frontal lobe and limbic system [2]. Approximately 50 million people are affected globally [3]. Unfortunately, no definitive treatments for AD or its progression have been identified, although temporary symptomatic therapies are available, with many side effects [4, 5]. Research into AD has advanced, particularly in neuroimaging, animal models, and biomarkers, though the exact molecular mechanisms remain unclear [1]. Multifunctional agents that target multiple biological pathways hold potential, and antioxidants, as well as sulfonamides, which possess neuroprotective and anti‐inflammatory properties, are promising candidates for future therapeutic development [4, 6].

The modern therapeutic approaches build upon the so‐called personalized medicine, which is defined as the tailoring of healthcare to individual patients through the integration of diagnostics, treatment, genetic testing, and advanced technologies such as proteomics and metabolomics. The primary advantages of this approach include enhanced treatment efficacy and reduced adverse effects, such as those linked to overdosing with drugs that have a narrow therapeutic index, like digoxin and anticoagulants [7]. Personalized medicine offers the potential to customize doses for each patient. Looking ahead, it would be valuable to develop an effective and safe system that allows remote and digital control by healthcare providers for improved patient management [8].

In this regard, 3D printing of pharmaceuticals could improve individualized pharmacotherapy through personalized dosages, enhancing both the safety and effectiveness of certain drugs. Various 3D printing technologies allow the production of solid dosage forms with different designs by layer‐by‐layer material deposition based on digital models. Pharmaceutical 3D printing in hospitals and pharmacies could enable the manufacture of tailored drug products, including multi‐active ingredient formulations, custom‐release profiles, and complex geometries, meeting all clinical needs. Fused deposition modeling (FDM) 3D printing offers unique opportunities for flexible, personalized drug production by depositing thermoplastic filament through a heated extrusion process. This technique, which does not involve powders or solvents, could be beneficial for low‐cost, on‐site drug manufacturing in clinical environments, as it avoids post‐processing and results in mechanically strong tablets. The key advantages of FDM printing include the absence of residual solvents, making it an environmentally friendly technology. However, challenges include the risk of thermal degradation of active pharmaceutical ingredients (APIs) or polymers during processing, and the limited processability depending on the mechanical properties of the filaments. FDM 3D‐printed tablets often exhibit slow dissolution due to the encapsulation of APIs in polymer matrices with erosion‐controlled release mechanisms. Numerous FDM formulations exhibit slow drug release, even when hydrophilic model drugs are incorporated into water‐soluble polymer matrices. For orally administered drugs with dissolution‐limited absorption, even a small increase in dissolution rate can significantly enhance bioavailability. The development of rapidly dissolving 3D printed tablets for lipophilic compounds is therefore crucial for the future production of personalized drug forms, as most drug candidates have poor water solubility [8, 9, 10].

The present study aims to develop, in line with the concept of personalized medicine, 3D‐printed tablets containing the recently synthesized novel sulfonamide derivatives with potential for the AD treatment. These promising candidates were first identified in our previous paper titled “New biologically active sulfonamides as potential drugs for Alzheimer's disease” [11]; the study was primarily focused on the synthesis and preliminary biological evaluation together with in vitro dissolution testing. Since the low aqueous solubility of these sulfonamides represents a limiting factor for their potential use as therapeutic agents for Alzheimer's disease, they were further converted into their hydrochloride salts, which exhibit higher solubility in aqueous solutions, consequently leading to improved bioavailability [11]. In the present paper, the alternative approach, i.e., the API amorphization within the polymer matrix produced via hot‐melt extrusion (HME), will be focused as the way to enhance the solubility of the poorly soluble crystalline API. The main advantage of this approach is its simultaneous consistence with the concept of personalized medicine, increasingly requested by nowadays pharmaceutical trends.

The present study introduces, as the title promises, a thorough guidance through the relevant characterization of the synthesized APIs, followed by the detailed description of the utilized HME and FDM processes (including the characterization of the prepared filaments and tablets), and concluded by the cytotoxicity and in vitro release (dissolution) tests. The specifically focused aspects of the present investigation are as follows: (1) Since a certain degree of antibacterial activity can be expected for the substances from the sulfonamide class, the antimicrobial activity of the present compounds is verified. (2) The reliable and non‐destructive conversion of the sulphonamide compounds to amorphous form is the second major goal of the present work. As HME involves processing polymeric materials above their glass transition temperature (T_g_) to achieve molecular‐level mixing of thermoplastic binders and/or polymers and active compounds, we utilize HME to prepare filaments based on AFFINISOL (hydroxypropyl methylcellulose) [12], a water‐soluble cellulosic polymer that enhances the solubility of poorly soluble drugs by stabilizing solid dispersions and preventing API crystallization. Thermo‐analytical characterization of the extruded filaments and FDM‐prepared tablets is used to confirm the amount of the amorphized API and to identify the temperature limits for the API processing. (3) The prepared filaments and tablets are subject to an all‐encompassing universal set of manipulation/storage‐related tests, i.e., the measurements of hardness, mechanical properties, moisture content, hygroscopicity, and disintegration. (4) Lastly (and most importantly), an extensive biorelevant testing of the prepared tablets containing the newly synthesized sulfonamides is performed: the in vitro dissolution testing, cytotoxicity, and stability tests. Accordingly, a thorough discussion is conducted over the comparison of the present dissolution profiles (obtained for the FDM‐produced tablets) and the profiles not only previously published [11] for the matrix tablets with the same APIs.

**FIGURE 1:**
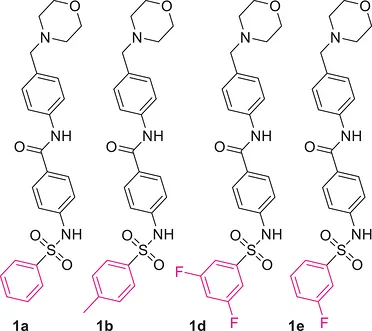
Structures of selected compounds.

Several promising candidates for the treatment of AD were identified, due to their biological activity (see Table 3), in our previous paper [11], as we mentioned above. Herein, four compounds were selected as our model drugs and were prepared in large quantities according to the previously published synthetic pathway [11]. The structures of these compounds are outlined in Figure 1.

## 2 Materials and Methods

### 2.1 Materials

All materials, reagents, and solvents were purchased from commercial sources (Sigma–Aldrich, Merck, Fluorochem, DuPont, Lachema, Penta, LachNer, Acros Organics, Gattefossé SAS, JPS Pharma GMBH & CO). Commercial‐grade reagents were used without further purification. The sulphonamides were synthesized as described in [11]; for the purpose of the present study, the APIs are denoted **1a**, **1b**, **1d**, and **1e** (note that in [11], these compounds were labeled 9a, 9b, 9d, and 9e, respectively).

### 2.2 Preparation of Filaments by HME Process

Studied filaments were prepared by hot‐melt extrusion process on a single‐screw extruder Noztek Touch (Noztek LTD, Shoreham‐by‐Sea, Great Britain). The width of the used nozzle was 1.75 mm.

Low glass transition HPMC suitable for the HME process, available under the trade name Affinisol HPMC HME 15LV, was selected as the basic component. In addition to the Affinisol powder, the input mixtures usually contained 10%, 20%, 30%, or 50% of one of the selected active compounds. The composition of extruded filaments/3D‐printed tablets together with the parameters of the HME process are shown in Table 1. The individual components were weighed and homogenized for 30 min in the V‐mixer (Filtra, Barcelona, Italy) to prepare the blend for hot‐melt extrusion. The filament was pulled out manually without using an automatic winder.

**TABLE 1:** The composition of prepared filaments/3D‐printed tablets with the parameters of the HME process (temperature and revolutions per minute). The numbers in parentheses (**1a‐1e**) refer to the selected APIs (see Figure 1 in the introduction).

Affinisol HPMC HME 15LV (mass %)	API (mass %)	HME process
90	10 (**1a**)	155°C, 25 rpm
80	20 (**1a**)	150°C, 25 rpm
70	30 (**1a**)	155°C, 25 rpm
90	10 (**1d**)	140°C, 24 rpm
90	10 (**1e**)	145°C, 24 rpm
80	20 (**1b**)	155°C, 30 rpm
50	50 (**1b**)	155°C, 28 rpm

### 2.3 Fused Deposition Modeling

Tablets designed in Autodesk Fusion 360 were exported into a 3mf file. The desired model of the 3D printed tablet was designed according to the previously published lipophilic and hydrophilic matrix tablets [11], which means a diameter of 13 mm and weight of 500 ± 10 mg. Only in the case of the filament with 50% of compound **1b** was weight 200 ± 10 mg. The height was adjusted for each filament composition due to filament thickness.

FDM 3D printers from Prusa Research company (i3 MK3S+) were used. The first step of the 3D printing process was a first layer (Z‐axis) calibration to obtain the optimal distance between the printing nozzle and the bed. One channel arrangement with a nozzle size of 0.4 mm was used. The whole process was kept under “complete individual objects” mode, whereas more objects out of the collision radius could be printed at once. Table 2 shows the most important 3D printing conditions.

**TABLE 2:** Summary of the most important 3D printing parameters.

3D printing parameter	Filaments with 1a, 1d, 1e	Filaments with 1b
T_bed_	60°C	60°C
T_nozzle_	195°C	195°C
Infill	80%	80%
Fill pattern	concentric	rectilinear
Perimeters	2	2
Extrusion multiplier	1	1
Layer height	0.2 mm	0.2 mm
Number of bottom solid layers	7	7

For one dissolution test, six tablets with incorporated active ingredient and one blank tablet were needed. The blank sample was represented by a tablet from pure Affinisol filament.

### 2.4 Thermal Analysis and Microscopy

Thermal stability of the prepared compounds, extruded filaments, and 3D‐printed tablets was determined via thermogravimetric (TGA) measurements, using the STA 449 F5 Jupiter instrument (Netzsch, Germany) equipped with a DSC/TG holder. The samples with masses in the order of mg (accurately weighted to ± 0.01 mg) were placed in open low‐mass Al pans and heated from 50°C to 550°C at 10°C·min^−1^. Two series of TGA experiments were performed—either in N_2_ or in a synthetic air atmosphere; the purge gas flow was always 50 mL·min^−1^. The second utilized thermoanalytical technique was the differential scanning calorimetry (DSC), which was used to monitor the form (crystalline vs. amorphous) of the compounds during the particular stages of their processing: synthesis → HME → FDM. In this regard, the Q2000 calorimeter (TA Instruments, USA) was used, equipped with an autosampler, RCS90 cooling accessory, and T‐zero technology. The DSC samples were, again, in the order of mg (accurately weighted to ± 0.01 mg), and the measurements were performed in hermetically sealed low‐mass Al pans (static air atmosphere). The applied temperature program was a single heating step at 10°C·min^−1^ in the 30°C–250°C temperature range. The third utilized thermoanalytical method was the dynamic mechanical analysis (DMA). The DMA 303 Eplexor instrument (Netzsch) was used in the 3‐point bending mode for the filaments, and in the compression/penetration mode in the case of the tablets. All measurements were performed at laboratory temperature (25°C), i.e., well below the first recognizable effects from the DSC measurements. During the 3‐point bending experiments, the distance between the support pins was 40 mm; the static force loaded in the middle of this distance was 0.2 N, further supplemented with a sinusoidal dynamic component—amplitude of 0.1 N and 1 Hz frequency; no static or dynamic strain limits were applied. The moduli were calculated for the cylindrical geometry corresponding to the true shape of the filaments. The mechanical resilience of the tablets was measured in the compression mode, using the penetration probe with a cylindrical pin (1 mm diameter); the experiments were conducted as a simple force ramp, i.e., via increasing the applied force from 0.1 to 50 N at 0.1 N·s^−1^. For each sample, two measurements with different points of contact were conducted: the point of contact was either in the middle of the top tablet base or in the middle of the lateral surface (with the tablet positioned on its edge). To supplement the thermal analysis, optical microscopy was used to visualize the homogeneity of the HME‐produced filaments; the micrographs were taken with the calibrated adjustable Dino‐Lite Edge 3.0 (AnMo Electronics Corporation, New Taipei City, Taiwan) microscopic probe in reflection mode, using deep focus processing.

### 2.5 Hardness

The C50 Tablet Hardness & Compression Tester (Engineering Systems, UK) was used to assess the hardness of the 3D‐printed tablets. Six measurements were taken for each tablet composition. The force in newtons was recorded. The results were expressed as mean values (N) ± SD.

### 2.6 Moisture Content

Moisture content (MC) expressed as a percentage in the samples was assayed in an HX204 halogen moisture analyzer (Mettler Toledo, Switzerland) under a drying temperature of 105°C. A total of 0.5 g of the sample (3D‐printed tablet) was used for each measurement, in total 3 measurements were taken. The results were expressed as mean values (%) ± SD.

### 2.7 Hygroscopicity

For the hygroscopicity test, 6 tablets were weighed and placed in a stability box (WTC Binder, Germany) at a constant temperature of 40°C and humidity of 75% RH. The tablets were weighed after 1, 2, 3, 7, 14, 30, and finally 60 days. The results were expressed as a percentage of weight gain.

### 2.8 Disintegration

For the disintegration test according to the *Ph. Eur*. at Erweka ZT500 instrument (Erweka GmbH, Germany), 6 tablets were used [13]. A disintegration test was performed with disks first in a pH 1.2 (0.1 m HCl) simulating gastric environment for 2 h, followed by pH 6.8 (phosphate buffer) simulating intestinal environment for the next 4 h. The volume of both media was 900 mL, temperature 37 ± 0.5°C. Disintegration time was monitored.

### 2.9 Cytotoxicity

Human hepatocellular carcinoma (HepG2, HB‐8065, ATCC) cells were cultured in Eagle's Minimum Essential Medium (EMEM, ATCC, Manassas, VA, USA). Neuroblastoma cell line (SH‐SY5Y, CRL‐2266, ATCC) was cultured in Dulbecco's Modified Eagle's Medium (DMEM, ATCC). Both were prepared with the addition of 10% fetal bovine serum (FBS; Gibco, Life Technologies, Waltham, MA, USA), 1% antibiotic mixture (penicillin and streptomycin), in standard culture conditions (5% CO_2_, 37°C, 95% humidity). The tested compounds were applied from DMSO stock solutions and diluted in the culture medium to the working concentrations. The influence of the DMSO on the viability of cells in the highest applied compound concentration was checked.

Cell viability was determined using 3‐(4,5‐dimethylthiazol‐2‐yl)‐2,5‐diphenyltetrazolium bromide (MTT) assay. Cells (HepG2, SH‐SY5Y) were seeded at a density of 1 × 10^4^ in 96‐well plates. After 24 h, cells were incubated with the tested compounds (**1a**, **1b** in their hydrochloride form) in concentrations of 1; 2; 5; 10; 25; 50 and 100 µm. After 24 or 48 h, 12 µL of MTT reagent was added to each well. After 2 h of incubation (37°C, 5% CO_2_), the medium was removed, and formazan produced in cells appeared as dark crystals at the bottom of the wells. Next, 100 µL of formazan dissolvent (DMSO) was added to each well. Then, the optical density of the obtained solution at 570 nm was determined using a plate reader (Spectra Max iD3, Molecular Devices, San Jose, CA, USA). Each individual experiment was repeated at least three times.

Data are expressed as mean ± SEM. Statistically significant values were compared using the Kruskal–Wallis and ANOVA tests using GraphPad Prism 5 software (GraphPad Software, San Diego, CA, USA); *p*‐values of less than 0.01/0.05 were considered statistically significant [21].

### 2.10 In Vitro Drug‐Release Studies

Sulfonamide release from 3D‐printed tablets was studied by the dissolution test method using a dissolution apparatus (Sotax AT 7 Smart, Allschwill, Switzerland). All dissolution tests were performed according to the *European Pharmacopeia* [13] in a paddle arrangement. Three different dissolution media prepared according to the *Ph. Eur*. were used: (1) an acidic medium of pH 1.2 (HCl with an adjustment of ionic strength using NaCl); (2) a duodenum medium of pH 4.5; (3) a colonic medium of pH 6.8 (KH_2_PO_4_ with NaOH). One dissolution test lasted 24 h with a stirring rate of 100 rpm. The bath temperature was maintained at 37 ± 0.5°C throughout the test. During the dissolution tests at predetermined time intervals, 3 mL aliquots of the dissolution medium were automatically withdrawn and filtered. Each experiment was performed with six tablets and one blank sample. In all dissolution tests, the released amount of the drug was determined using UV–vis spectrometry, and the dissolution profiles obtained were evaluated using suitable kinetic models [13].

### 2.11 Determination of the Drug‐Release Amount Using UV–Vis Spectrometry

An HP Agilent 8453 spectrophotometer (Agilent Technologies, Santa Clara, CA, USA) was used to determine the selected drugs' concentration in the dissolution samples. The absorbance values of the samples withdrawn at the predetermined times were measured against the corresponding blank sample using the fixed‐wavelength method, together with a three‐point background correction. The used wavelengths corresponded to the absorption maximum of the selected drugs in an appropriate pH. The calibration‐curve method was used to transform the absorbance values into concentrations and percentages. These percentages were plotted against time to obtain the dissolution profile of the drug.

The obtained dissolution profiles were fitted to the Weibull (1) and the first‐order kinetic (2) models expressed by the following equations:

(1) $$M_{t}=M_{\infty }\;\left(1-\exp\left(-k_{w}t^{\beta }\right)\right)\;$$

(2) $$M_{t}=M_{\infty }\;\left(1-\exp\left(-k_{1}t\right)\right)$$

where *M_t_* is the amount of the drug released in time *t*, *M_∞_* is the maximum releasable amount of the drug in infinite time, *k_w_* is a constant of the Weibull model with unit *time* ^−^ *^β^*, and *k_1_* is a first‐order rate constant with unit *time* ^−^ *^1^* [14, 15]. GraphPad Prism 9.3.1 (GraphPad Software, San Diego, CA, USA) was employed for the non‐linear regression analysis [22].

### 2.12 Stability

For stability testing, 6 tablets of the selected compositions were subjected to a dissolution test after the end of the hygroscopicity test, which means after 60 days in a stability box (WTC Binder, Germany) at a constant temperature of 40°C and humidity of 75% RH.

## 3 Results and Discussion

### 3.1 Biological Activity

In our previously published study [11], we performed in vitro evaluation of acetylcholinesterase‐(AChE) and butyrylcholinesterase‐(BChE) inhibiting activity of new sulfonamides via Ellman's method [16]. The results were compared to those for the standard rivastigmine (RIV), a cholinesterase inhibitor employed in the treatment of dementia related to Alzheimer's and Parkinson's diseases. The inhibitory activity was expressed as IC_50_, representing the concentration of the inhibitor causing 50% inhibition of the enzyme. Here in Table 3, we show the activity only of selected compounds for an overview.

**TABLE 3:** In vitro AChE and BChE inhibition of selected compounds (IC_50_ (µm)) compared with that of standard rivastigmine (RIV). Cholinesterase inhibition is expressed as the mean ± SD (*n* = 3 experiments).

Compounds as hydrochloride salts	IC_50_ (µm)	
	AChE	BChE
**1a**	10.83 ± 0.36	104.98 ± 7.06
**1b**	7.95 ± 0.43	30.17 ± 1.17
**1d**	17.13 ± 0.99	> 500
**1e**	14.64 ± 0.09	138.31 ± 1.60
**RIV**	56.10 ± 1.41	38.40 ± 1.97

As might be expected, selected compounds showed antimicrobial activity [17]. The minimum inhibitory concentration (MIC) was determined by the microdilution method according to EN ISO 20776‐1 ED [18]. Table 4 summarises the activity of selected compounds. The most pronounced antimicrobial effects were shown by compounds **1a** and **1b**. The pronounced antibacterial activity—particularly against *Enterococcus faecalis*—although not the primary focus of this work, raises concerns regarding oral administration. For these compounds, alternative routes of delivery (e.g., local administration) should therefore be considered, or further structural modifications pursued to reduce antimicrobial off‐target effects associated with the selected molecules.

**TABLE 4:** In vitro antimicrobial activity of selected compounds in their hydrochloride form (MIC (mg/L)) compared with that of standard sulfamethoxazole.

Microorganism	MIC (mg/L)				
	1a	1b	1d	1e	Sulfamethoxazole
*Candida albicans* (CCM 8215)	> 32	> 32	> 32	> 32	> 32
*Candida glabrata* (CCM 8270)	1	1	4	> 32	> 32
*Candida krusei* (CCM 8271)	1	1	> 32	16	> 32
*Candida tropicalis* (CCM 8223)	32	> 32	> 32	> 32	> 32
*Enterococcus faecalis* (CCM 4224)	1	1	8	1	1
*Enterococcus faecalis* (VRE) (isolate)	1	1	1	1	1
*Escherichia coli* (CCM 3954)	> 32	> 32	> 32	> 32	32
*Pseudomonas aeruginosa* (CCM 3955)	> 32	> 32	> 32	> 32	16
*Staphylococcus aureus* (CCM 2022)	> 32	> 32	> 32	> 32	> 32

### 3.2 Preparation of Filaments by HME Process

In total, 7 filaments with incorporated selected active compounds were successfully prepared by the hot melt extrusion process on the single‐screw extruder Noztek Touch. The temperatures of this process were moving from 140°C to 155°C. The composition of these filaments, together with properties such as diameter of filaments and also 3D‐printed tablets, is summarized in Table 5. Complete amorphization of the API was confirmed in four of the prepared filaments, while residual crystalline fractions were detected in the remaining three cases (see Section 3.4). Stabilizing molecules in an amorphous state using a solvent‐free process (HME + FDM) appears to be a suitable approach, as the majority of newly developed drug molecules exhibit poor aqueous solubility [23, 24, 25]. A range of drug loadings was successfully achieved, with the filament containing 50% of compound **1b** representing a notable success. Tablets were successfully printed from all prepared filaments.

**TABLE 5:** The composition and properties of prepared filaments/3D‐printed tablets. The numbers in parentheses (**1a‐1e**) refer to the selected APIs (see Figure 1).

Affinisol HPMC HME 15LV (mass %)	API (mass %)	Diameter of filaments (mm)	Diameter of 3D‐printed tablets (mm)
90	10 (**1a**)	1.77	12.92
80	20 (**1a**)	1.80	13.34
70	30 (**1a**)	1.79	12.96
90	10 (**1d**)	1.82	12.97
90	10 (**1e**)	1.83	12.89
80	20 (**1b**)	1.54	12.76
50	50 (**1b**)	1.55	12.83

To date, only a limited number of researchers have successfully incorporated high API concentrations (above 40 wt.%) into filaments, as printability—particularly mechanical stability and flexibility—tends to decrease with increasing particle loading [26].

### 3.3 Fused Deposition Modeling

**FIGURE 2:**
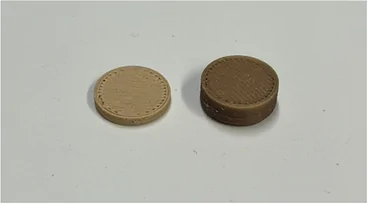
3D‐printed tablets containing 50% of compound 1b (left); 20% of compound 1a (right).

In an effort to improve solubility and the dissolution rate of prepared sulfonamides, we have focused on the amorphization process. To ensure amorphization of selected compounds, the temperature of the 3D printing process was set slightly higher than the melting temperature of relevant compounds. The printing temperature was set at 195°C. A similar approach was employed by Manini et al. in their study on the amorphization of paliperidone palmitate [24]. 3D printed tablets were successfully printed on FDM 3D printers from Prusa Research company (i3 MK3S+). Selected 3D printed tablets are shown in Figure 2. As you can see, the resulting 3D‐printed tablets appear darker than the original filaments due to the higher processing temperature during the second thermal process—FDM. Nevertheless, the thermal stability of the matrix was confirmed in our previous publication up to 220°C, which exceeds the experimental temperatures applied in this study [12]. Both formulations containing compound **1b** still exhibited detectable amounts of the crystalline phase. It was used a concentric filling pattern in the case of compounds **1a**, **1d** and **1e**, and a rectilinear filling pattern in the case of compound **1b** to provide better mechanical properties.

In their study, Gioumouxouzis et al. [27] reported only partial incorporation of the originally crystalline metformin API into the amorphous dispersion. Okwuosa and colleagues [28] highlighted the potential of dual‐nozzle FDM 3D printing; XRPD analysis indicated the presence of theophylline crystals in the 3D‐printed tablets, whereas budesonide and diclofenac remained in the amorphous state. Jamróz et al. [29] confirmed the amorphization of aripiprazole in 3D‐printed tablets, whereas traces of the crystalline form were still detectable in the filaments.

### 3.4 Thermo‐Analytical Characterization

**FIGURE 3:**
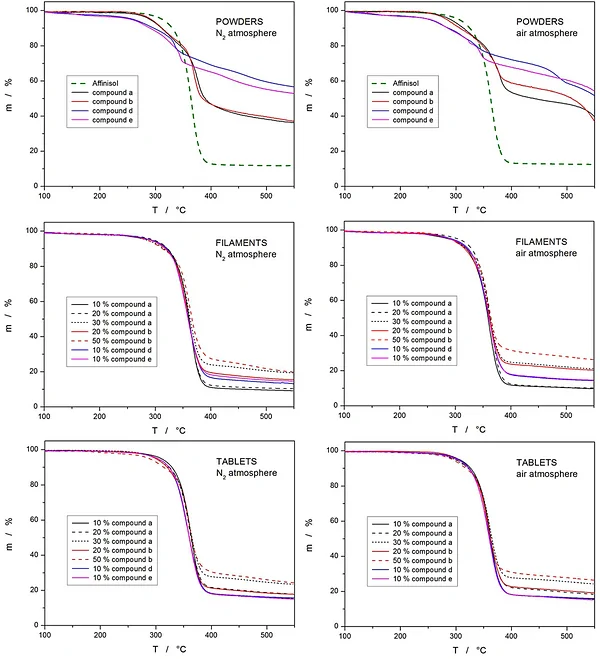
TGA curves obtained at 10°C·min^−1^ for the studied substances (in their powder form), filaments and tablets.

From the thermo‐analytical point of view, the most important aspect of the newly synthesized drugs is their thermal stability during the HME and FDM processes. In this regard, the TGA data are the most relevant ones. In Figure 3, the comparison of the mass loss signals obtained for the pure APIs and filaments and tablets containing these APIs is shown. Before commenting on the present data, it is worth noting that the main benchmark is the decomposition behavior of pure Affinisol, which was found [12] to be stable up to ∼ 260°C under N_2_ atmosphere but exhibited slow partial decomposition (oxygen‐catalysed split of the hydroxypropyl and methyl groups) near 150°C in the air environment. Hence, all APIs intended for the production of the HME/FDM‐prepared Affinisol‐based dosage forms should be stable at least up to this temperature. This is indeed the case for the **1a** and **1b** compounds. However, the **1d** and **1e** APIs exhibit a ∼ 2% mass loss being initiated already at T < 120°C. Whereas this mass loss is most probably associated with the release of certain volatiles and/or synthesis by‐products, it has to be born in mind that further purification of these APIs may be needed (in comparison with the procedures presented here). The TGA data measured for the prepared filaments and tablets are fairly uniform, since the minor low‐T mass loss processes already fully proceeded during the corresponding processing (HME at 140°C–155°C, and FDM at 195°C). The residual masses well correspond to the contents and thermal stabilities of the individual APIs.

**FIGURE 4:**
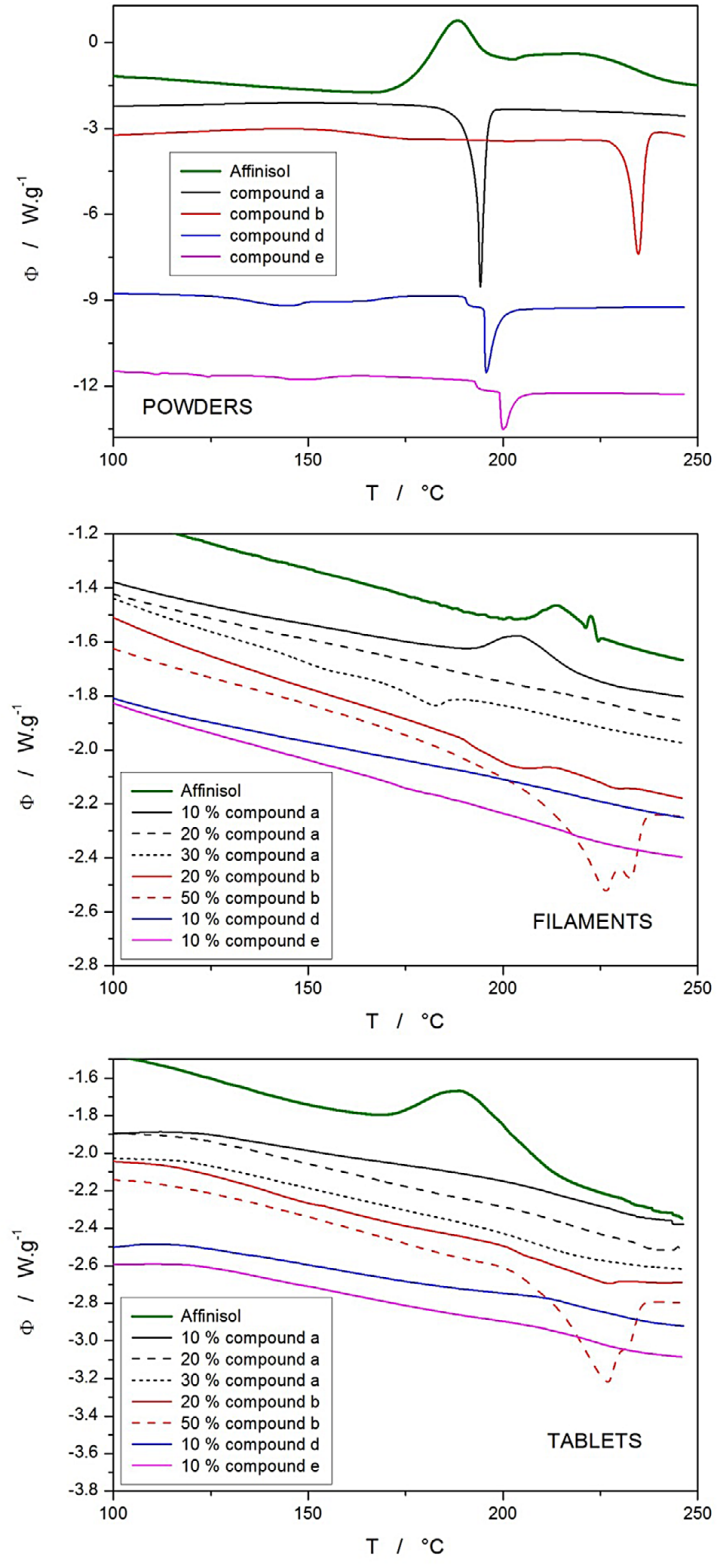
DSC curves obtained at 10°C·min^−1^ for the studied substances (in their powder form), filaments and tablets (the data are compared to a placebo filament and tablet prepared from pure Affinisol, with no API contained). Exothermic effects evolve in the upward direction.

Further insight into the low‐T thermo‐kinetic behavior of the present APIs and corresponding filaments and tablets was provided through DSC—see Figure 4. Starting with the pure Affinisol powder, a characteristic double‐peak exothermic signal occurs between 170°C and 240°C, corresponding to the partial split of the hydroxypropyl and methyl groups [12]. Each pure API then exhibits a characteristic endothermic melting peak with the following extrapolated onset temperatures T_m_ and melting enthalpies ΔH_m_: **1a** (T_m_ = 193.1°C, 112.5 J·g^−1^), **1b** (T_m_ = 231.8°C, 94.5 J·g^−1^), **1d** (T_m_ = 195.2°C, 62.4 J·g^−1^), **1e** (T_m_ = 199.0°C, 42.8 J·g^−1^). Apart from the melting peaks, several other minor endothermic signals were recorded for certain APIs, probably indicating the release of volatiles and impurities from the synthesis (as already suggested by the TGA data). The DSC measurements of filaments and tablets containing the present APIs were performed with two main goals in mind—first, to assess the state of the API (crystalline vs. amorphous) within the given product based on the presence of the corresponding melting peak, and second, to consider the possible interactions between the API and the Affinisol matrix (based on the presence of the exothermic decomposition peak). Note that the exothermic decomposition signal is clearly present for both filaments and tablets from pure Affinisol (although distorted due to the previous HME/FDM thermal treatment), thus its absence indicates an interaction between the hydroxypropylmethylcellulose (HPMC) functional side‐groups and the contained API.

**FIGURE 5:**
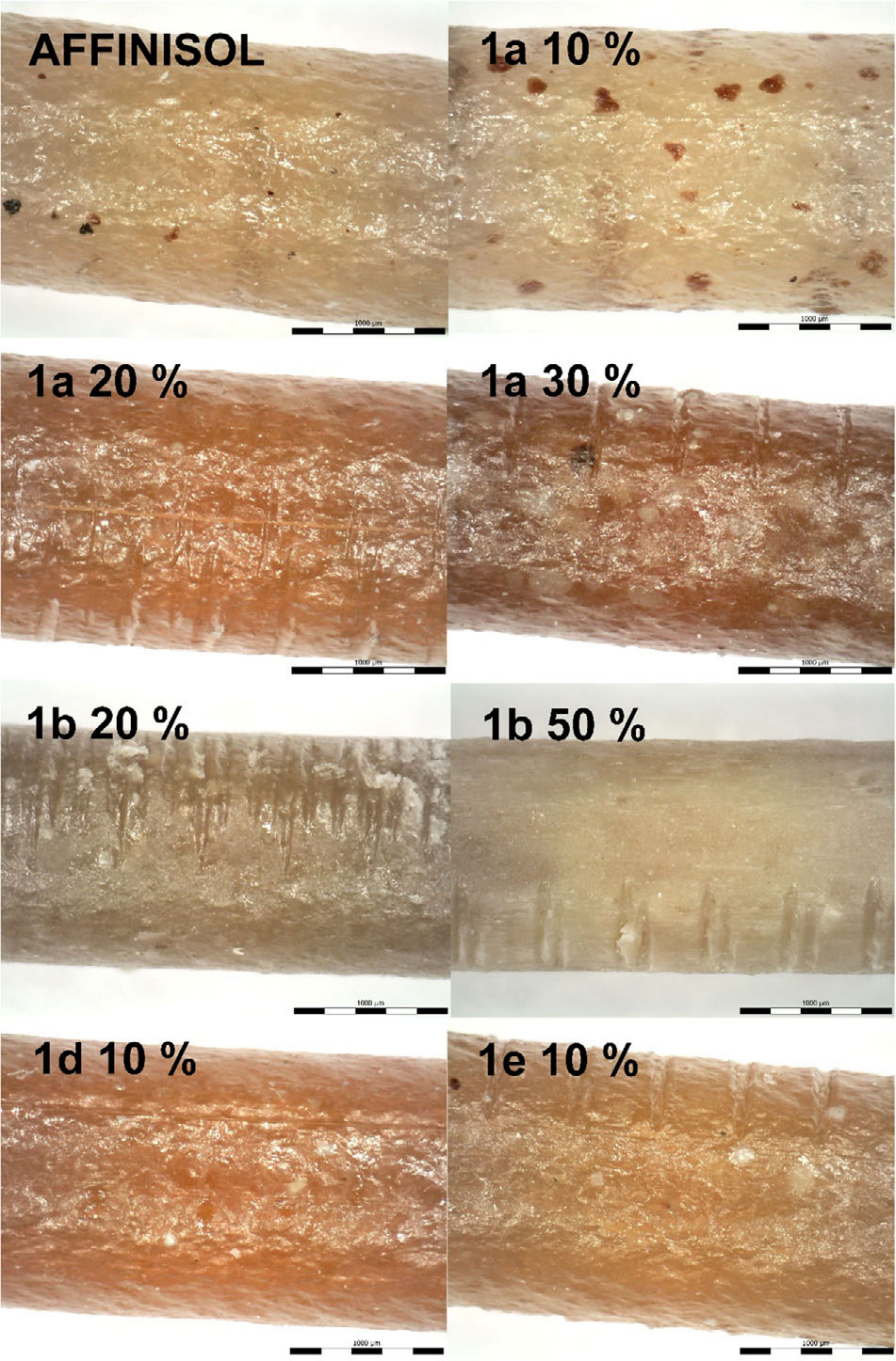
Optical micrographs of the prepared filaments. The scale bar indicates 1 mm.

The DSC data obtained for the filaments (see Figure 4) indicate that in the majority of cases (except for 10% of the **1a** API being dispersed in the HPMC filament), the API content, distribution, and activity are sufficient to ensure the sheathing of (interaction with) a majority of the HPMC side‐groups. On the contrary, in several cases, the content of the API is high enough to exceed the saturation of the API‐HPMC interaction that results in API amorphization. The exceeding amount of API remains in the crystalline form, which can be detected via the corresponding melting signal. These were indeed detected in the following cases: filament with 30% **1a** (approx. 6% crystalline), filament with 20% **1b** (approx. 8% crystalline), filament with 50% **1b** (approx. 39% crystalline), tablet with 20% **1b** (approx. 10% crystalline), tablet with 50% **1b** (approx. 40% crystalline). These results are, however, only rough estimates, as the shapes and positions of the melting peaks changed considerably and, thus, certain interactions can also be expected here. Note that the content of the crystalline API can ultimately be interpreted in terms of the maximum uptake of the synthesized compounds by the HPMC matrix. To further explore this idea, optical micrographs of the extruded filaments were taken—see Figure 5. As expected, in the case of the above‐mentioned samples (30% of **1a**, 20% of **1b**, and 50% of **1b**), light‐colored inhomogeneities are present within the filament's volume, which probably corresponds to the clustered reservoirs of the crystalline (not fully dissolved/dispersed) API. Note that the dark‐colored spots visible in the case of the pure Affinisol and 10% of **1a** filaments are a rather common [12, 19] finding for the Affinisol‐based matrices, and possibly indicate certain overheated spots with larger amounts of the polymer matrix being degraded.

**FIGURE 6:**
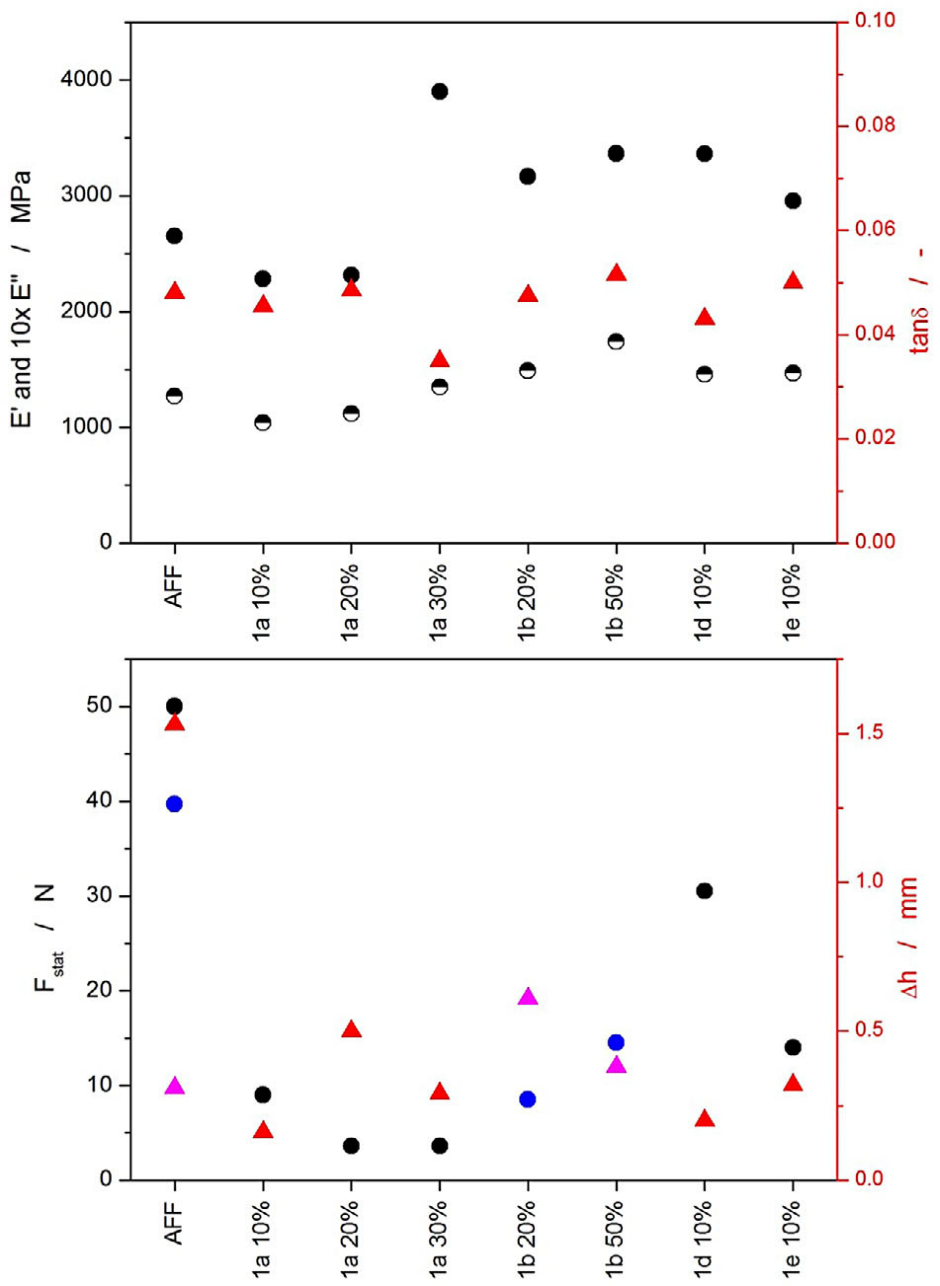
Top graph—storage modulus E’, loss modulus E’’ and loss factor tanδ obtained for the filaments during the 3‐point bending test. Bottom graph –indentation depth Δh during the compressive penetration of the top tablet base, and the critical load F_stat_ at which the tablet layers detached during the compressive penetration applied to the tablet edge/side. The blue/magenta datapoints correspond to the tablets with rectilinear filling pattern (AFF tablets with both filling patterns were tested).

In addition to the monitoring of the mass loss and heat flow, mechanical properties relevant to the FDM procedure as well as to the consequent manipulation with the tablets (i.e., data measured at 25°C) were determined—see Figure 6. The 3‐point bending test performed for the extruded filaments has shown that all materials are highly elastic (tanδ ≈ 0.05); apart from the filaments containing 10% and 20% of **1a** compound, all other API‐containing filaments exhibited higher stiffness and toughness in comparison with the pure Affinisol filament. The printed tablets were subject to the two penetration tests: (1) during the spot‐compressive force being applied to the top tablet side, all formulations withstood the full F_stat_ = 50 N load without rupture or penetration, while the maximum achieved deformations Δh (see Figure 6) were significantly lower (by approx. 2/3) compared to that of the pure Affinisol tablet. This again testifies about the considerably higher toughness of the API‐containing HPMC matrices; note, however, that the pure AFF tablet printed out with the rectilinear filling pattern exhibited similarly high toughness, which is otherwise achieved via API impact on the printing process. (2) When the spot‐compression was applied to the tablet edge/side, only the pure Affinisol tablet printed with concentric filling pattern withstood the full 50 N load (the AFF tabled printed with rectilinear filling pattern withstood only ∼ 40 N, which is, however, still significantly higher than the API‐loaded formulations). For the other tablets, a separation of the printed layers occurred at relatively low loads (F_stat_ values depicted in Figure 6) as a consequence of the API in‐mixing and predefined printing parameters.

### 3.5 Physico‐Chemical Properties

Among the four evaluated candidates, only the two most promising, due to their biological activity (see Table 3), thermo‐analytical properties, and a range of prepared filaments with varying compositional percentages (see Table 5), were selected for subsequent testing. The chosen compounds (**1a**, **1b**) in the composition of 20% active compound + 80% of Affinisol were submitted to tests like hardness, moisture content, hygroscopicity, and disintegration. The results are summarised in Table 6 below.

**TABLE 6:** Physico‐chemical properties of 3D printed tablets. Presented results are expressed as mean value ± SD.

Physico‐chemical test	1a	1b
Hardness (N)	77.2 ± 1.1	34.3 ± 6.5
Moisture content (%)	0.36 ± 0.17	0.97 ± 0.19
Hygroscopicity (%)	6.11 ± 0.68	6.79 ± 0.24
Disintegration (min)	> 360	> 360

As we can observe in Table 6, there are some differences between the results. The cause can probably be retrieved by the type of filling pattern, a concentric filling pattern by **1a** and a rectilinear filling pattern by **1b**, which we assumed would provide better physicochemical properties. Contrary to expectations, and in line with the thermo‐analytical characterization above, tablets printed with a concentric filling pattern exhibit approximately double the hardness. Additionally, the moisture content is much lower with a concentric filling pattern. Nevertheless, the type of filling pattern appeared to have no significant effect on either hygroscopicity or disintegration. After 60 days (40°C, 75% RH), the hygroscopicity in both cases exceeded 6%. In the disintegration test, all 6 tablets lasted over 6 h in both formulations.

### 3.6 Cytotoxicity

**FIGURE 7:**
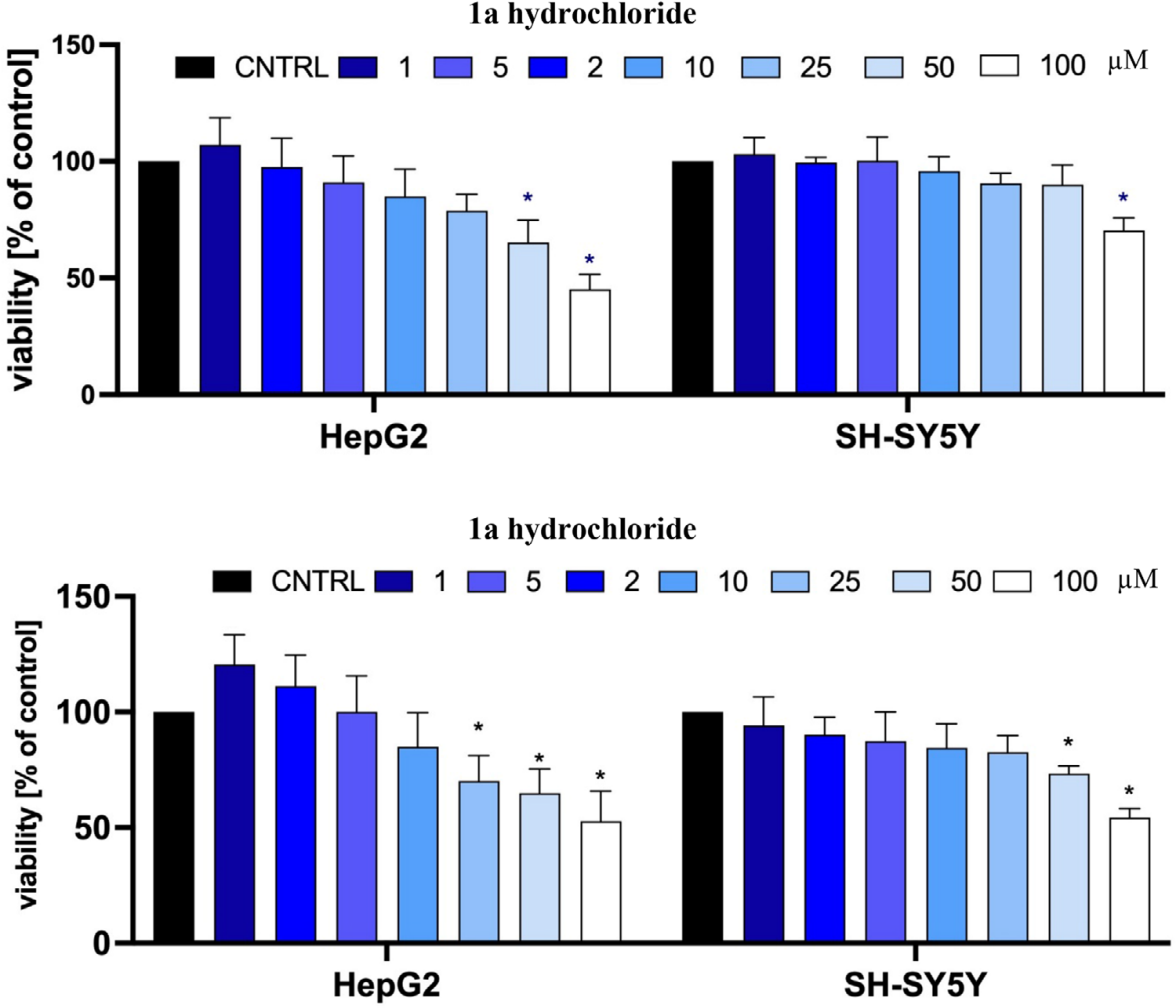
Cytotoxicity assay of compound 1a as hydrochloride salt on two cell lines: HepG2 and SH‐SY5Y after 24 h (up) and 48 h (down) of incubation.

**FIGURE 8:**
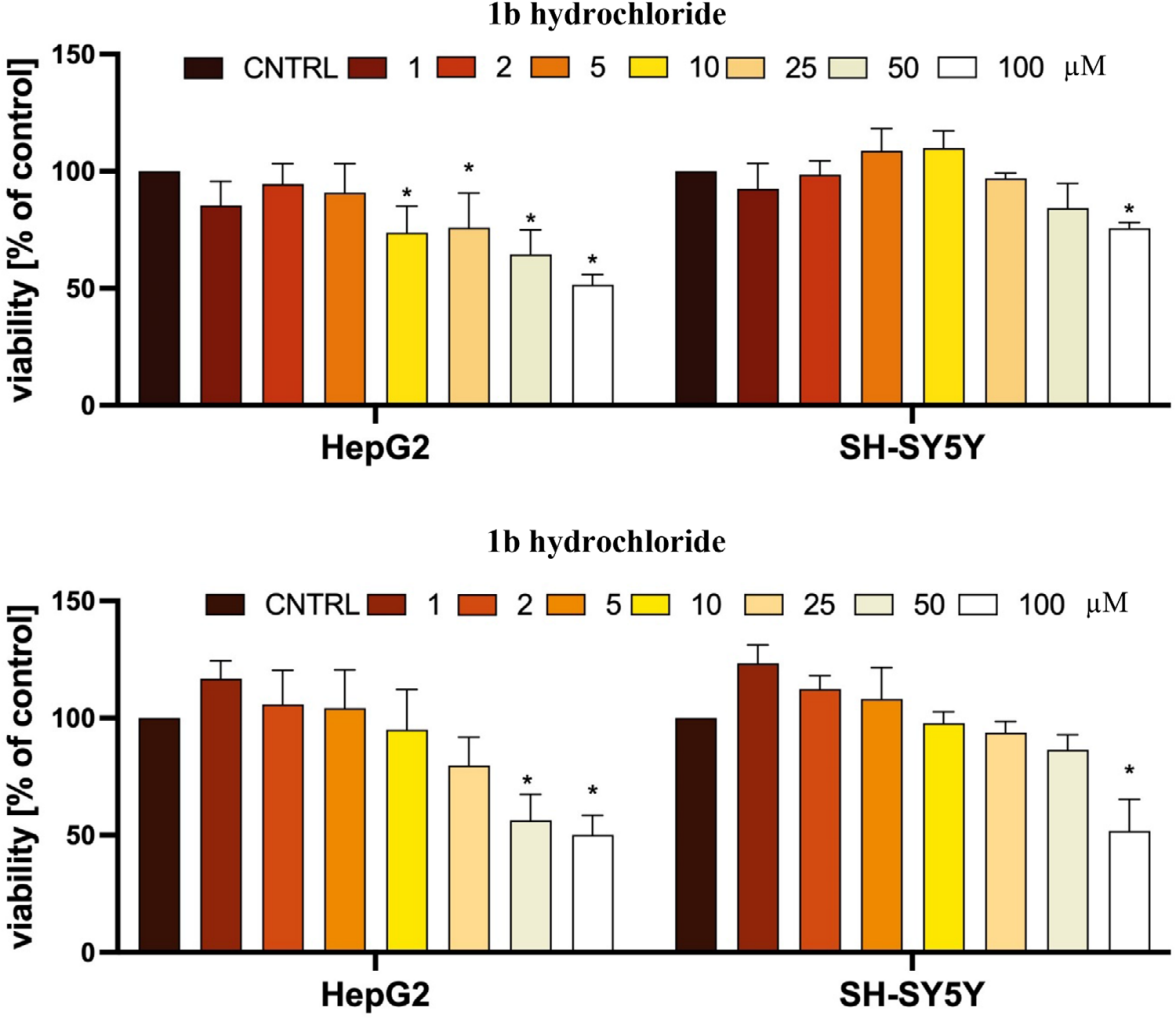
Cytotoxicity assay of compound 1b as hydrochloride salt on two cell lines: HepG2 and SH‐SY5Y after 24 h (up) and 48 h (down) of incubation.

To assess the effect of compounds **1a** and **1b** in their hydrochloride form on the viability of HepG2 hepatocellular carcinoma cells and SH‐SY5Y human neuroblastoma cells, a 24‐ and 48‐h incubation with a wide concentration range (1–100 µm) of the compounds was performed, followed by an MTT assay. The MTT assay allows the evaluation of cellular metabolic activity (mitochondrial enzyme function), which under strictly controlled experimental conditions correlates with cell viability. The control samples consist solely of cells (HepG2, SH‐SY5Y) without the addition of active pharmaceutical ingredients. HepG2 cells showed greater sensitivity to the tested compounds compared to SH‐SY5Y cells (see Figures 7 and 8). Compound **1a** exhibited a cytotoxic effect only at the highest concentrations (50 and 100 µm) after 24 h of incubation. In contrast, compound **1b** significantly reduced the viability of HepG2 cells already at a concentration of 10 µm, whereas SH‐SY5Y cells remained largely resistant to its action (cytotoxicity was observed only at the highest tested concentration). After 48 h of incubation, compound **1a** caused a reduction in the viability of HepG2 cells at a concentration of 25 µM, whereas SH‐SY5Y cells remained less sensitive, with a significant decrease in viability observed only at the highest concentrations (50 and 100 µm). Compound **1b**, after 48 h of incubation, reduced the viability of HepG2 cells at the highest tested concentrations, while SH‐SY5Y cells showed a noticeable reduction in viability only at 100 µm. To gain deeper theoretical insight into their pharmacokinetic disposition, we performed an *in silico* ADME profiling using the SwissADME predictor for the newly developed derivatives (compounds **1a** and **1b**), evaluated in their neutral free‐base form. The BOILED‐Egg model (see Figure S1) predicted high gastrointestinal absorption; however, due to the calculated physicochemical parameters, the compounds were positioned outside the passive blood‐brain barrier (BBB) permeation zone. Since *in silico* tools strictly predict passive diffusion based on basic molecular descriptors, they cannot account for carrier‐mediated transport. Given the structural features of our derivatives, active influx mechanisms might be involved.

A comparison of cytotoxicity with the inhibitory activity of the selected compounds (IC_50_ for AChE of 10.83 for compound **1a** and 7.95 for compound **1b**) indicates only a narrow therapeutic index. The selected molecules serve primarily as model drug candidates to enable processing by HME and FDM 3D printing, and to explore strategies for enhancing the dissolution rate of poorly soluble compounds. The narrow therapeutic window precludes oral systemic administration. As an alternative strategy, local drug delivery (bypassing first‐pass hepatic toxicity) may be considered, or further structural modifications could be pursued to achieve higher inhibitory potency combined with reduced cytotoxicity.

### 3.7 In Vitro Drug Release

**FIGURE 9:**
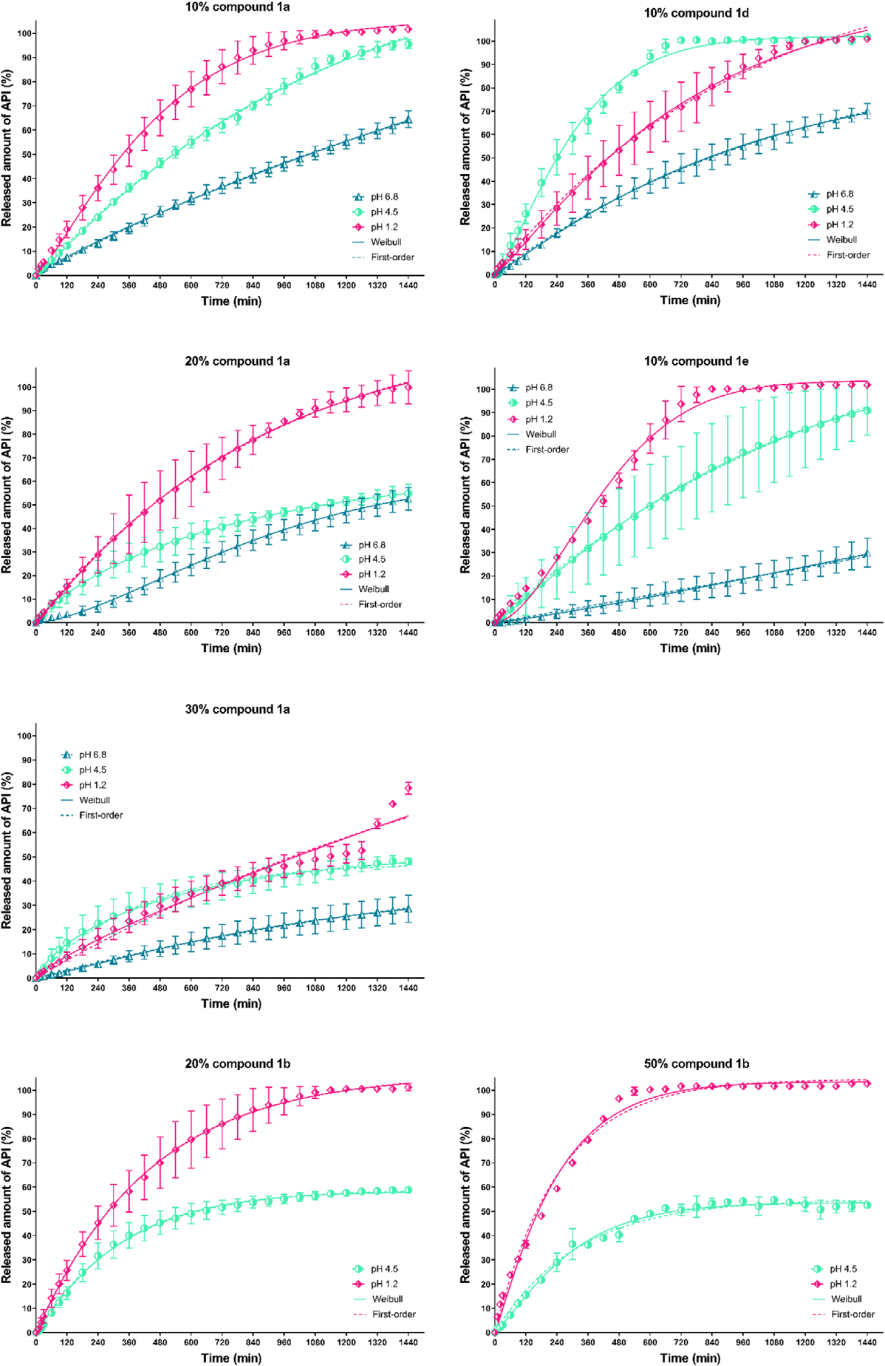
The dissolution profiles of the studied 3D‐printed tablets fitted to the first‐order and Weibull models. Error bars represent the standard deviation of the drug released (*n* = 6). Error bars for some points are smaller than the symbol.

The dissolution profile of the drug is one of the most important characteristics of the dosage form. A series of dissolution tests was performed to study the dissolution behavior of all prepared 3D‐printed tablets. The obtained dissolution profiles were quantitatively evaluated using appropriate mathematical models (the first‐order kinetic model and the Weibull model) [14, 15]. The regression analysis of the dissolution profiles is summarized in Table S2. The β parameter of the Weibull model (see Table S2) characterizes the shape of the dissolution curve. In most cases, the β value is close to 1, which is typical of an exponential release profile. Exceptions include the 10% compound **1a** at pH 1.2, 20% **1a** at pH 6.8, 10% **1d** at pH 4.5, and 10% **1e** at pH 1.2, where β exceeds 1 and a sigmoid‐shaped profile can therefore be observed (the release begins slowly, then accelerates, and subsequently decelerates again). In several cases (20% **1a** at pH 4.5 and 30% **1a** at pH 1.2 and 4.5), the β parameter was below 1, indicating a parabolic profile (an initial burst effect followed by a deceleration phase). The influence of pH and composition of 3D‐printed tablets were studied. The dissolution behavior of the studied 3D printed tablets is summarized in Figure 9.

Dissolution tests were conducted in dissolution media with pH values of 1.2, 4.5, and 6.8. The medium of pH 6.8 was omitted only for compound **1b** due to its poor solubility. As illustrated in Figure 9, the dissolution profiles of compound **1b** at 20% and 50% concentrations are very similar. Compound **1b** exhibited the highest amount released in the medium of pH 1.2.

In contrast, for compound **1a**, distinct dissolution curves were observed at 10%, 20%, and 30% concentrations, respectively. Similarly, compound **1a** showed the highest release of the active substance in the medium at pH 1.2 across all three concentrations. The observed decrease in dissolution from 10% to 30% is likely attributable to non‐compliance with the sink condition [20]. However, the objective was to demonstrate that a 30% filament could be prepared, from which tablets could be printed and subsequently subjected to dissolution testing.

Compound **1d** demonstrated the highest amount released in the medium of pH 4.5, while compound **1e** showed the highest release of API in pH 1.2 (see Figure 9). Notably, the addition of a second fluorine substituent in compound **1d** leads to distinct release profiles in comparison with compound **1e**, which contains only one fluorine substituent. Despite the observed variations, both compounds achieved greater than 90% release at the end of the dissolution study in both media (pH 1.2 and 4.5). These two compounds were utilized to extend the study.

Nevertheless, we consider the successful preparation of the 50% filament (compound **1b**), the successful printing of tablets, and the acquisition of dissolution profiles similar to those observed with the 20% filament/tablets as a partial success.

Although Patel and Serajjudin [30] prepared filaments with 40% and 50% drug loading, these were too brittle to be used for 3D printing. In contrast, Gioumouxouzis et al. [27] successfully produced a filament containing 50% metformin, from which they printed a bilayer dosage form together with an additional filament incorporating glimepiride.

### 3.8 Stability Testing

**FIGURE 10:**
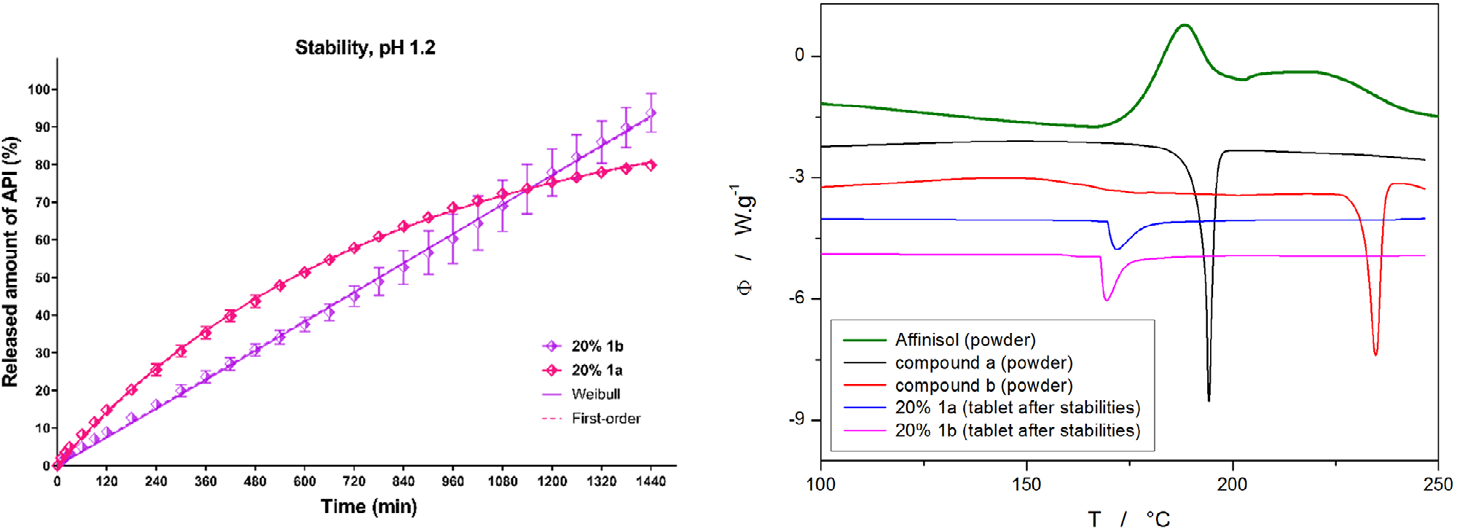
Left: The dissolution profiles of the two selected compositions after stability testing (60 days, 40°C, 75% RH) fitted to the first‐order and Weibull models. Error bars represent the standard deviation of the drug released (*n* = 6). Error bars for some points are smaller than the symbol. Right: Comparison of the DSC curves obtained for the powdered Affinisol, powdered pure compound 1a, powdered pure compound 1b, tablet containing 20% of compound 1a exposed to the stability testing, and tablet containing 20% of compound 1b exposed to the stability testing.

The dissolution test in pH 1.2 with two selected compositions was performed again after storage for 60 days at a temperature of 40°C and 75% RH. The resulting dissolution profiles are shown in Figure 10. As can be observed, both formulations result in the release of 80% or more of the API. Notably, compound **1b** exhibits zero‐order release kinetics, with its dissolution profile after stability testing appearing to differ significantly from the initial one. An increase in deviations over time can be observed, which may indicate a gradual degradation of the dosage form. To gain further insight into the markedly changed dissolution behavior of the two tablets subject to the stability testing, DSC measurements were performed—see Figure 10 (right graph). It was found that 100% of compound **1a** and ∼ 85% of compound **1b** were transformed into crystalline form during the stability testing (based on the comparison of the melting enthalpies, assuming that the newly formed crystalline phase has identical thermal properties as the originally synthesized APIs), which explains the significantly slower dissolution of both samples. The most probable cause for the APIs re‐crystallization is the plasticization of Affinisol, where the increased mobility within the system promotes the formation of the thermodynamically stable phase. It is also worth noting that the onset of the melting peaks has shifted to significantly lower temperature (corresponding to the onset of the oxidative thermal degradation of the Affinisol material), which indicates that the amorphous APIs re‐crystallized into either different (metastable) polymorphic forms, nanocrystalline material, or hydrates of the original sulfonamide compounds.

Similarly, Patel and Serajuddin [30], using HME and FDM, converted haloperidol into its amorphous form. In contrast, under the same conditions (40°C, 75% RH) but with half the duration (30 days), the DSC scans remained unchanged; however, it remains uncertain whether the scans would remain stable after 60 days. Fanous et al. [8] reported that lumefantrine in a 3D‐printed dosage form containing 5% API remained fully stable for 16 weeks post‐manufacture. Manini et al. [24] demonstrated the stability of amorphous paliperidone palmitate in 3D‐printed dosage forms at 4°C and 25°C over a period of 3 months.

### 3.9 Comparison of 3D‐Printed and Matrix Tablets

**FIGURE 11:**
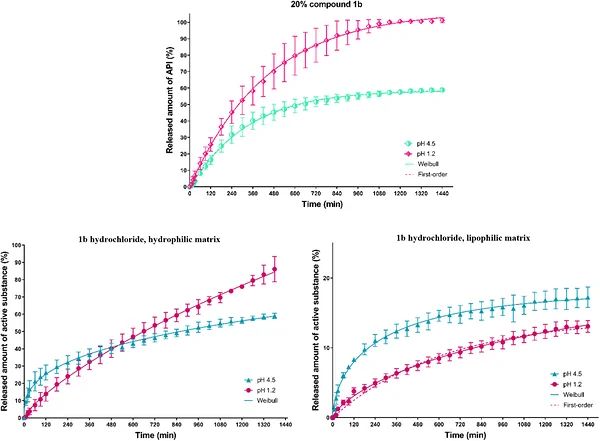
The comparison of 3D‐printed tablets (top) with both hydrophilic and lipophilic matrix tablets (bottom) with compound **1b**, respectively **1b** as hydrochloride salt.

**FIGURE 12:**
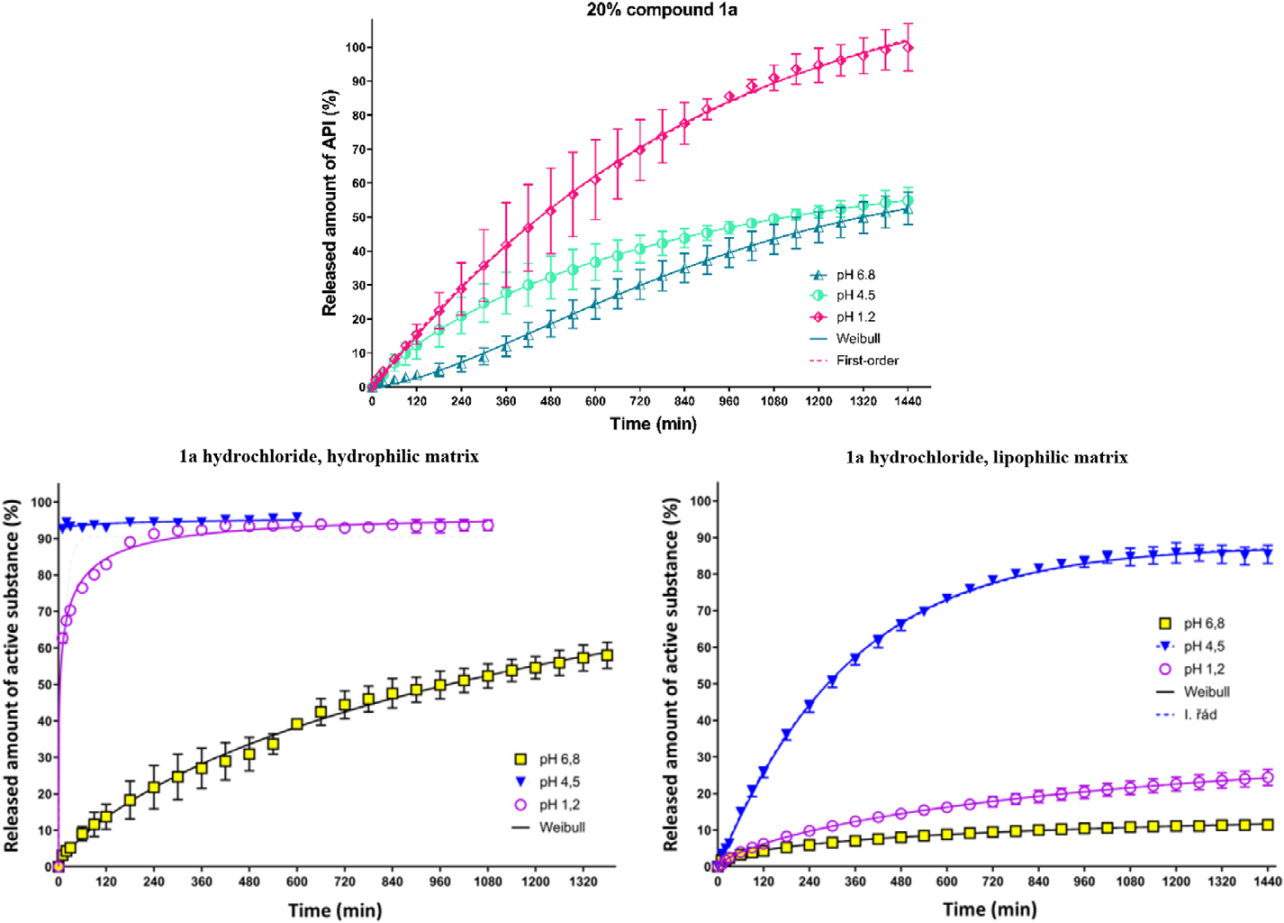
The comparison of 3D‐printed tablets (top) with both hydrophilic and lipophilic matrix tablets (bottom) with compound **1a**, respectively **1a** as hydrochloride salt.

Some dissolution profiles obtained in this study were compared with those of the same API in the form of matrix tablets, including both hydrophilic and lipophilic matrix tablets [11]. Moreover, the dissolution profiles of matrix tablets containing compound **1a** in its hydrochloride form were not included in the aforementioned previous publication, but were part of additional testing. The composition of these matrix tablets is in Table S1. The same substances were used in the form of salt (hydrochloride) for matrix tablets. These dissolution profiles are summarized in Figures 11 and 12.

As evident from the dissolution profiles, the dissolution curves differ both in the comparison between lipophilic and hydrophilic matrices and between matrix‐based and 3D‐printed tablets. Compound **1b** (respectively **1b** as hydrochloride salt) demonstrates favorable release in the dissolution medium at pH 1.2 when formulated as 3D‐printed tablets and hydrophilic matrix tablets. Similarly, compound **1a** (respectively **1a** as hydrochloride salt) exhibits optimal release at pH 1.2 under the same conditions; however, in the case of hydrophilic matrix tablets, a very rapid release of the entire API is observed, which may not be entirely desirable. Compound **1a** (respectively **1a** as hydrochloride form) also shows a favorable release profile at pH 4.5 when incorporated into lipophilic matrix tablets. Nevertheless, pretty good dissolution profiles are consistently achieved with 3D‐printed tablets (see Figures 11 and 12).

In an akin research, Hwang et al. [23] observed a faster release from certain amorphous forms of celecoxib prepared by HME combined with FDM, compared with a commercial formulation containing the crystalline form of the API. Similarly, Kimura et al. [31] demonstrated an improvement in the solubility of itraconazole in its amorphous state relative to the crystalline form, which was practically insoluble. Higher dissolution rates in 3D‐printed films were also confirmed by the group of Lee et al. [32], who investigated the incorporation and amorphization of aripiprazole.

## 4 Conclusion

In this study, seven filaments incorporating varying concentrations of selected sulfonamide derivatives were successfully prepared using HME, demonstrating their potential for application in personalized pharmaceutical manufacturing. All filaments exhibited sufficient printability, enabling the fabrication of solid dosage forms via FDM 3D printing. Notably, even filaments with high drug loading (up to 50% API) showed considerable success in terms of processability and performance.

Thermal and physicochemical analyses confirmed the thermal stability of the formulations and revealed molecular‐level interactions between the incorporated APIs and the Affinisol‐based polymer matrix. Importantly, successful amorphization of the incorporated APIs was observed in most 3D‐printed tablets, which may be critical for improving solubility and bioavailability. An exception was compound **1b**, which exhibited incomplete amorphization, particularly at higher loadings.

Cytotoxicity testing using HepG2 and SH‐SY5Y cell lines confirmed the safety of the two most promising candidates, supporting their further development. Extensive in vitro dissolution testing of the 3D‐printed tablets revealed multiple favorable release profiles, with several formulations demonstrating sustained or enhanced drug release suitable for the therapeutic requirements of Alzheimer's disease. These profiles remained favorable, despite the drug re‐crystallization, following preliminary storage stability studies.

When compared with conventional hydrophilic and lipophilic matrix tablets, the 3D‐printed tablets generally offered superior or comparable dissolution characteristics. The ability to fine‐tune API content, dosage form geometry, and print parameters offers a high degree of flexibility, underscoring the advantages of 3D printing, which may be applied further in achieving personalized drug delivery. Future work should focus on scaling these systems for clinical use with clinically used drugs and further elucidating the long‐term stability and in vivo performance of these novel dosage forms [33].

## Author Contributions

**Marie Nevyhoštěná**: Conceptualization, methodology, investigation, data curation, Writing – original draft, Writing – review and editing. **Alena Komersová**: Methodology, supervision, funding acquisition, writing – original draft, writing – review and editing. **Roman Svoboda**: Methodology, investigation, data curation, writing – original draft, writing – review and editing; **Vladimír Pejchal**: Investigation. **Adéla Pospíšilová**: Methodology and investigation; **Jan Muselík**: Supervision. **Aleksandra Łapa**: investigation and data curation. **Paulina Koczurkiewicz – Adamczyk**: Methodology, investigation, and writing – original draft.

## Funding

This work was supported by the Internal Grant Agency of the University of Pardubice under the SGS project (SGS_2026_006).

## Consent

All authors agreed with the journal policy and provided our consent for the publication.

## Conflicts of Interest

The authors declare no conflicts of interest.

## Supporting information




**Supporting File 1**: mabi70266‐sup‐0001‐SuppMat.tif.

**Supporting File 2**: mabi70266‐sup‐0002‐SuppMat.docx.

**Supporting File 3**: mabi70266‐sup‐0003‐SuppMat.docx.

## Data Availability

The data that support the findings of this study are available from the corresponding author upon reasonable request.
