# Supplementary material for: Personalized 3D‐Printed Affinisol‐Based Pharmaceutical Delivery Systems: The Case Study for Alternative Sulfonamides for Alzheimer's Disease
Source: Macromol Biosci. 2026 Sep 25;26(9):e70266. doi: 10.1002/mabi.70266 (PMC13615160; doi:10.1002/mabi.70266)
Supplement: Supplementary file 1 [file MABI-26-e70266-s002.docx]

Supplementary Material:

Table S1 The composition of the matrix tablets, total weight 500 ± 5 mg.

| **Hydrophilic matrix** | **Lipophilic matrix** | **Mass (%)** |
| --- | --- | --- |
| Prosolv^®^ SMCC 90 | Prosolv^®^ SMCC 90 | **39** |
| API | API | **20** |
| Methocel^TM^ K4M | Compritol^®^ 888 ATO | **40** |
| Magnesium stearate | Magnesium stearate | **1** |

*Table S2 Regression analysis of the dissolution profiles.*

|  | | | | **First-order:** $\boldsymbol{M}_{\boldsymbol{t}}\boldsymbol{=}\boldsymbol{M}_{\boldsymbol{\infty}}\left( \boldsymbol{1-}\exp\left( \boldsymbol{-}\boldsymbol{k}_{\boldsymbol{1}}\boldsymbol{t} \right) \right)$ | | | | | |  |  |
| --- | --- | --- | --- | --- | --- | --- | --- | --- | --- | --- | --- |
|  |  |  |  | $\boldsymbol{k}_{\boldsymbol{1}}$ **± SD [hour ^-1^]** | | | | **R^2^** | |  |  |
| pH 1.2 | | **20% compound 1b** | | 0.002261 ± 0.000207 | | | | 0.9789 | |  |  |
| pH 4.5 | |  |  | 0.002979 ± 0.000233 | | | | 0.9876 | |  |  |
| pH 1.2 | | **50% compound 1b** | | 0.004037 ± 0.00022 | | | | 0.9931 | |  |  |
| pH 4.5 | |  |  | 0.003169 ± 0.000267 | | | | 0.9838 | |  |  |
| pH 1.2 | | **10% compound 1a** | | 0.001769 ± 0.000119 | | | | 0.9837 | |  |  |
| pH 4.5 | |  |  | 0.000795 ± 0.000073 | | | | 0.9965 | |  |  |
| pH 6.8 | |  |  | 0.0004391 ± 0.000094 | | | | 0.9902 | |  |  |
| pH 1.2 | | **20% compound 1a** | | 0.001084 ± 0.000187 | | | | 0.9810 | |  |  |
| pH 4.5 | |  |  | 0.001686 ± 0.000216 | | | | 0.9694 | |  |  |
| pH 6.8 | |  |  | 0.0001385 ± 0.000141 | | | | 0.9684 | |  |  |
| pH 1.2 | | **30% compound 1a** | | 0.0004524 ± 0.000228 | | | | 0.9509 | |  |  |
| pH 4.5 | |  |  | 0.002446 ± 0.000438 | | | | 0.9516 | |  |  |
| pH 6.8 | |  |  | 0.00053 ± 0.000312 | | | | 0.9045 | |  |  |
| pH 1.2 | | **10% compound 1d** | | 0.001051 ± 0.000173 | | | | 0.9831 | |  |  |
| pH 4.5 | |  |  | 0.002846 ± 0.000188 | | | | 0.9855 | |  |  |
| pH 6.8 | |  |  | 0.0008413 ± 0.000147 | | | | 0.9869 | |  |  |
| pH 1.2 | | **10% compound 1e** | | 0.001519 ± 0.000227 | | | | 0.9750 | |  |  |
| pH 4.5 | |  |  | 0.0007137 ± 0.00034 | | | | 0.8627 | |  |  |
| pH 6.8 | |  |  | 0.000001264 ± 0.0000001 | | | | 0.8814 | |  |  |
|  | | | | **Weibull:** $\boldsymbol{M}_{\boldsymbol{t}}\boldsymbol{=}\boldsymbol{M}_{\boldsymbol{\infty}}\left( \boldsymbol{1}\boldsymbol{-}\exp\left( \boldsymbol{-}\boldsymbol{k}_{\boldsymbol{w}}\boldsymbol{t}^{\boldsymbol{\beta}} \right) \right)$ | | | | | | |  |
|  |  |  |  | $\boldsymbol{k}_{\boldsymbol{w}}$ **± SD [hour ^-β^]** | | **β ± SD** | | | **R^2^** | |  |
| pH 1.2 | **20% compound 1b** | | | 0.002124 ± 0.00062 | | 1.012 ± 0.054 | | | 0.9789 | |  |
| pH 4.5 |  |  |  | 0.002237 ± 0.00059 | | 1.054 ± 0.048 | | | 0.9879 | |  |
| pH 1.2 | **50% compound 1b** | | | 0.002281 ± 0.00044 | | 1.106 ± 0.036 | | | 0.9941 | |  |
| pH 4.5 |  |  |  | 0.001095 ± 0.00034 | | 1.194 ± 0.055 | | | 0.9874 | |  |
| pH 1.2 | **10% compound 1a** | | | 0.00052 ± 0.00012 | | 1.224 ± 0.039 | | | 0.9869 | |  |
| pH 4.5 |  |  |  | 0.000597 ± 0.000099 | | 1.069 ± 0.036 | | | 0.9967 | |  |
| pH 6.8 |  |  |  | 0.0003901 ± 0.00016 | | 0.9035 ± 0.0566 | | | 0.9905 | |  |
| pH 1.2 | **20% compound 1a** | | | 0.0008769 ± 0.00035 | | 1.045 ± 0.082 | | | 0.9810 | |  |
| pH 4.5 |  |  |  | 0.003434 ± 0.00095 | | 0.8488 ± 0.0637 | | | 0.9713 | |  |
| pH 6.8 |  |  |  | 0.000032 ± 0.000019 | | 1.508 ± 0.1095 | | | 0.9744 | |  |
| pH 1.2 | **30% compound 1a** | | | 0.0000047 ± 0.001497 | | 0.8037 ± 0.1238 | | | 0.9559 | |  |
| pH 4.5 |  |  |  | 0.006774 ± 0.002666 | | 0.7931 ± 0.0857 | | | 0.9560 | |  |
| pH 6.8 |  |  |  | 0.0002862 ± 0.000247 | | 1.165 ± 0.182 | | | 0.9054 | |  |
| pH 1.2 | **10% compound 1d** | | | 0.0005654 ± 0.00023 | | 1.127 ± 0.078 | | | 0.9839 | |  |
| pH 4.5 |  |  |  | 0.0005378 ± 0.000117 | | 1.300 ± 0.038 | | | 0.9927 | |  |
| pH 6.8 |  |  |  | 0.0006034 ± 0.000205 | | 1.077 ± 0.072 | | | 0.9872 | |  |
| pH 1.2 | **10% compound 1e** | | | 0.00002604 ± 0.00001 | | 1.708 ± 0.068 | | | 0.9926 | |  |
| pH 4.5 |  |  |  | 0.0004634 ± 0.000394 | | 1.105 ± 0.181 | | | 0.8631 | |  |
| pH 6.8 |  |  |  | 0.0000142 ± 0.000194 | | 1.141 ± 0.292 | | | 0.8850 | |  |
|  | | |  | |  | |  | | | | |
|  | | |  | |  | |  | | | | |
|  | | |  |  |  | |  | | | | |

*Figure S1 BOILED-Egg model calculated for compounds* ***1a*** *and* ***1b****.*
