# Supplementary material for: Personalized 3D‐Printed Affinisol‐Based Pharmaceutical Delivery Systems: The Case Study for Alternative Sulfonamides for Alzheimer's Disease
Source: Macromol Biosci. 2026 Sep 25;26(9):e70266. doi: 10.1002/mabi.70266 (PMC13615160; doi:10.1002/mabi.70266)
Supplement: Supplementary file 2 [file MABI-26-e70266-s003.docx]

**Highlights:**

- Seven drug-loaded filaments were successfully prepared and 3D-printed using HME and FDM.
- Most sulfonamide APIs were amorphized in tablets, enhancing potential bioavailability.
- Dissolution profiles of tablets remained favorable and stable, suitable for Alzheimer's therapy.
- 3D printing enabled highly flexible, personalized dosage forms superior to conventional tablets.
